# Septic Embolism Associated With Periodontal Disease: A Case Report

**DOI:** 10.7759/cureus.40468

**Published:** 2023-06-15

**Authors:** Diana V Castro, Simone Costa, Odete Gomes, Nuno Ferreira, Luís Pereira

**Affiliations:** 1 Intensive Care Unit, Centro Hospitalar de Leiria, Leiria, PRT

**Keywords:** bacteremia, periodontal disease, septic embolism, provetella oralis, parvimonas micra

## Abstract

*Parvimonas micra* and *Provetella oralis* are two commensal anaerobic bacteria of the human oral cavity. Anaerobic bacteria infections are uncommon and require a high index of suspicion and a quick start of appropriate treatment. We present a patient with multifocal infiltrates compatible with septic embolism (lung, liver, and spleen emboli) and polymicrobial bacteremia with *Parvimonas micra* and *Provetella oralis*. Periodontal disease appears to be the main cause of this disseminated infection.

## Introduction

*Parvimonas micra* and *Provetella oralis* are two anaerobic bacteria that are part of the human commensal flora [1]. *Parvimonas micra* is a gram-positive anaerobic coccus that colonizes the oral cavity and gastrointestinal tract and is mainly recognized as an oral pathogen often isolated in periodontitis and abscesses [2-4]. *Provetella oralis* is a gram-negative anaerobic bacteria of the oral flora, and *Provetella* species are among the dominant microflora isolated from secondary dental caries [5]. Periodontal disease, including periodontitis, has been reported to be a rare cause of septic embolism. Precipitating factors include malignancies, immune deficiencies, chronic renal disease, decubitus ulcers, and perforated abdominal viscera [6].

## Case presentation

A 31-year-old man, with a past medical history of congenital deafness and not on any medication, presented to the emergency room with fatigue and generalized weakness after one week of evolution. This was associated with vomitting, diarrhea, anorexia, asthenia, and weight loss (six kilos in two weeks). He denied fever, cough, chest pain, abdominal pain, hematochezia, hematemesis, constipation, or changes in the urinary tract. He denied any travel or any other kind of exposure. He refers to the occasional consumption of smoked cocaine and denies snorting cocaine or other intravenous drug use. The patient’s blood pressure at the time of admission was 112/60mmHg, heart rate of 130 beats per minute in sinus rhythm, respiratory rate of 27/minute, and a blood oxygen saturation of 95% on room air. His tympanic temperature was 39.5ºC. Physical examination revealed pale and sweaty skin as well as pain on palpation of the upper abdominal quadrants. The neurological examination was completely unremarkable. The patient had bad oral hygiene and evidence of periodontal disease.

**Figure 1:**
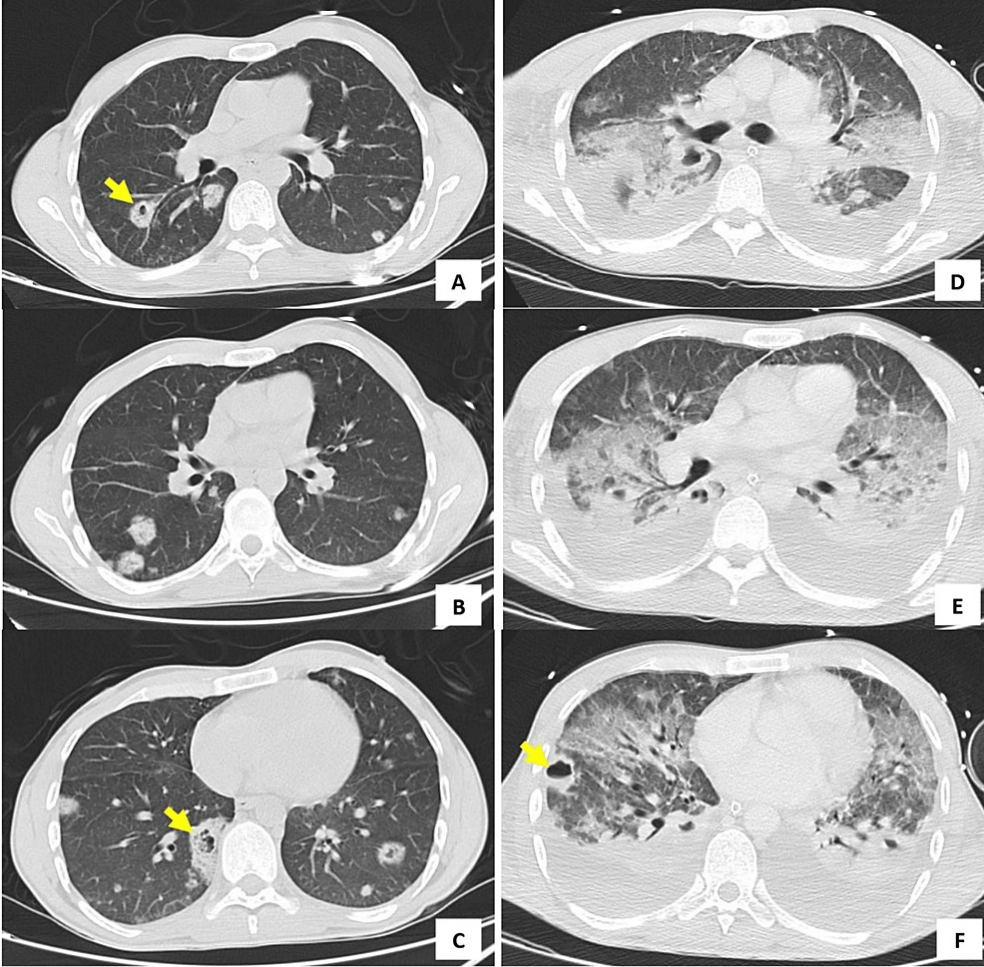
A CT thoracic scan shows septic pulmonary emboli at hospital admission (panel A, B, C) and at intensive care admission one week later (panel D, E, F). The arrows show pulmonary cavities.

**Figure 2:**
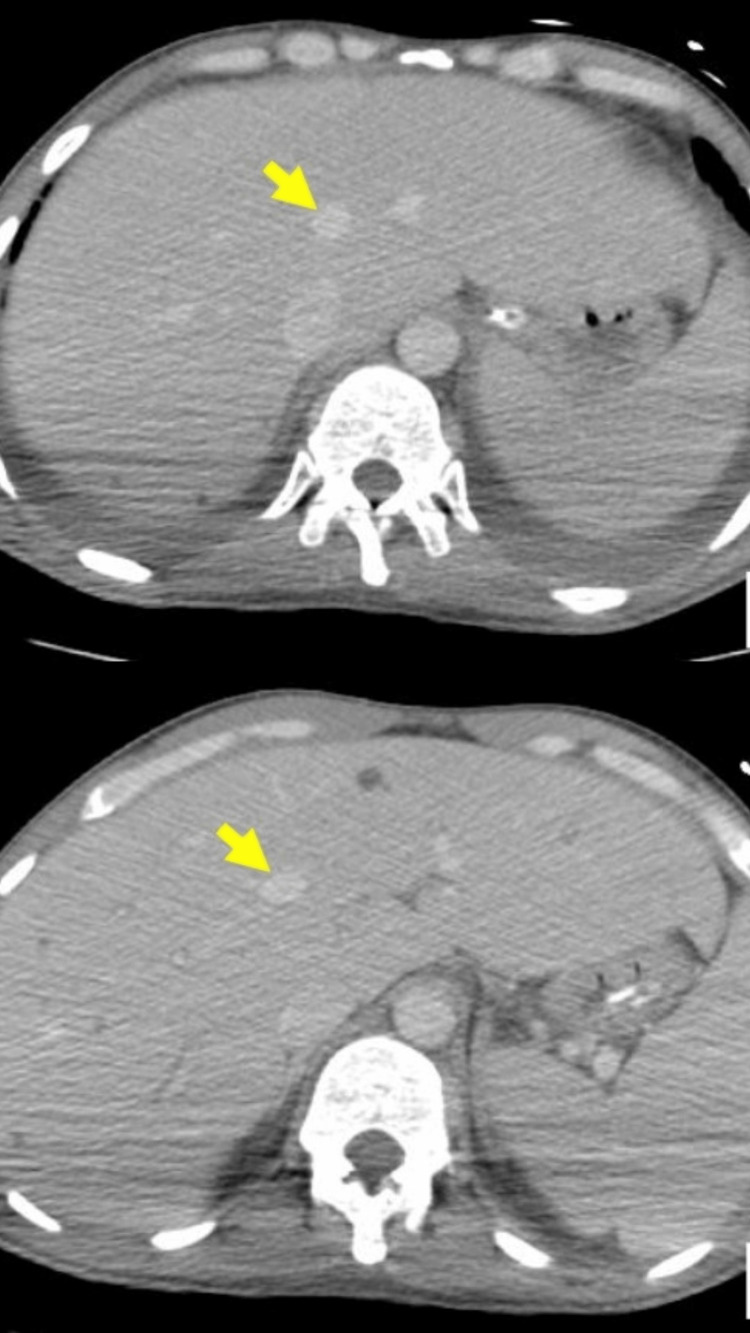
An abdominal scan shows septic hepatic emboli. The arrows show microabscesses in the liver (panel A and B).

Laboratory studies revealed a C-reactive protein of 155mg/dL (normal <5mg/L) without leukocytosis, severe thrombocytopenia of 3000 uL, and an acute kidney injury with a creatinine of 1.12mg/dL (normal 0.51-0.95mg/dL). Hepatitis B and C virus (HBV and HBC) and human immunodeficiency virus (HIV) serological tests were negative. Contrast-enhanced computed tomography (CT) of the thorax, abdomen, and pelvis revealed several areas of pulmonary parenchyma densification with central cavitation suggestive of septic emboli and hypodense images suggestive of microabscesses in the liver and spleen (Figure 1 and 2).

Sputum and blood culture reports were ordered, and then intravenous antibiotics with ceftriaxone and vancomycin were started. On the third day of hospital stay, the patient's condition got worse with dyspnea and increased work of breathing, requiring intensive care unit (ICU) admission and the start of mechanical ventilation. In two blood cultures, *Parvimonas micra* and *Prevotella oralis* were isolated, and antibiotic therapy was directed to meropenem. Transesophageal echocardiography was performed and showed the absence of heart valve vegetation. The cranial CT scan was also normal.

When the patient was extubated after ten days of mechanical ventilation, dental medicine was called and identified dental caries. The extraction of three dental pieces was performed. A maxillofacial CT scan excluded any abscesses. Treatment with meropenem was maintained for three weeks. The patient was discharged from ICU after twelve days, and it was confirmed that there was a complete remission of lung, hepatic, and spleen abscesses by CT scan, and there were no symptoms of recurrence. At 28 days of follow-up, the patient remained clinically stable, and laboratory findings were normal. Blood cultures were performed, and they were negative. The remaining study of immunodeficiencies is ongoing.

## Discussion

*Parvimonas micra* and *Provetella oralis* are two anaerobic bacteria that are associated with periodontal disease [1,2,5]. In this case report, there are multifocal infiltrates compatible with septic embolism, mainly in the lung parenchyma but also in the liver and spleen. Other potential causes, like endocarditis, were excluded, and periodontal disease appears to be the main cause.

In the literature, osteoarticular infections like spondylodiscitis are the preferred location of the infection, followed by heart valves, pleura, and multifocal infiltrates with bacteremia, as in this case report, are rare [4,6,7]. We found only four case reports of bacteremia due to *Parvimonas micra*, all with specific circumstances: one patient with esophageal cancer [2]; one patient after endoscopic retrograde cholangiopancreatography [8]; one patient with colonic carcinoma [6]; and another patient with septic pulmonary embolism [9]. We also found a few cases of *Provetella oralis* bacteremia. Due to the severity and mortality rate of anaerobic bacteremia, antibiotic therapy must be promptly started, and any identified abscess must be drained to achieve source control. These two anaerobic bacteria are usually susceptible to metronidazole, but, although resistances to this antibiotic are rare, resistant strains have been reported [5,10,11]. Septic embolism, in particular pulmonary embolism, is usually due to gram-positive organisms. Methicillin-sensitive *Staphylococcus aureus* (MSSA) (28%) and methicillin-resistant *Staphylococcus aureus* (MRSA) (16%) account for the majority of patients [5,9,10]. Therefore, the initial choice of the antibiotic regimen was ceftriaxone and vancomycin, although this patient didn’t have any risk factors for multi-resistant bacteria like MRSA. As the clinical condition of the patient didn´t improve and required mechanical ventilation, antibiotic therapy was switched to meropenem despite the identification of microorganisms. Regarding *Provetella oralis*, a gram-negative anaerobic bacteria, fluroquinolones are often used, but increasing resistance has been documented. With rising resistance to fluoroquinolones by *Provetella oralis*, clinicians should be aware of clinical failure in patients taking these antimicrobials and switch to meropenem, another considered regimen for this agent [1,12].

## Conclusions

Infections caused by anaerobic bacteria are rare and require high suspicion to diagnose and a quick start of treatment. Isolation of the causative pathogen is rare for periodontal disease because these infections are usually polymicrobial. The treatment of choice has not yet been established, but it must be noted that resistance to metronidazole is not uncommon. It is also important to control the source, and if an abcess is identified, surgical drainage is mandatory.
